# Correction: A dual photoredox-nickel strategy for remote functionalization *via* iminyl radicals: radical ring-opening-arylation, -vinylation and -alkylation cascades

**DOI:** 10.1039/c9sc90192b

**Published:** 2019-09-26

**Authors:** Elizabeth M. Dauncey, Shashikant U. Dighe, James J. Douglas, Daniele Leonori

**Affiliations:** School of Chemistry, University of Manchester Oxford Road Manchester M13 9PL UK daniele.leonori@manchester.ac.uk; Early Chemical Development, Pharmaceutical Sciences, AstraZeneca, R&D Macclesfield SK10 2NA UK

## Abstract

Correction for ‘A dual photoredox-nickel strategy for remote functionalization *via* iminyl radicals: radical ring-opening-arylation, -vinylation and -alkylation cascades’ by Elizabeth M. Dauncey *et al.*, *Chem. Sci.*, 2019, **10**, 7728–7733.

The authors regret that Scheme 1 did not appear correctly in the original article. The correct version of Scheme 1 is presented below.

**Scheme 1:**
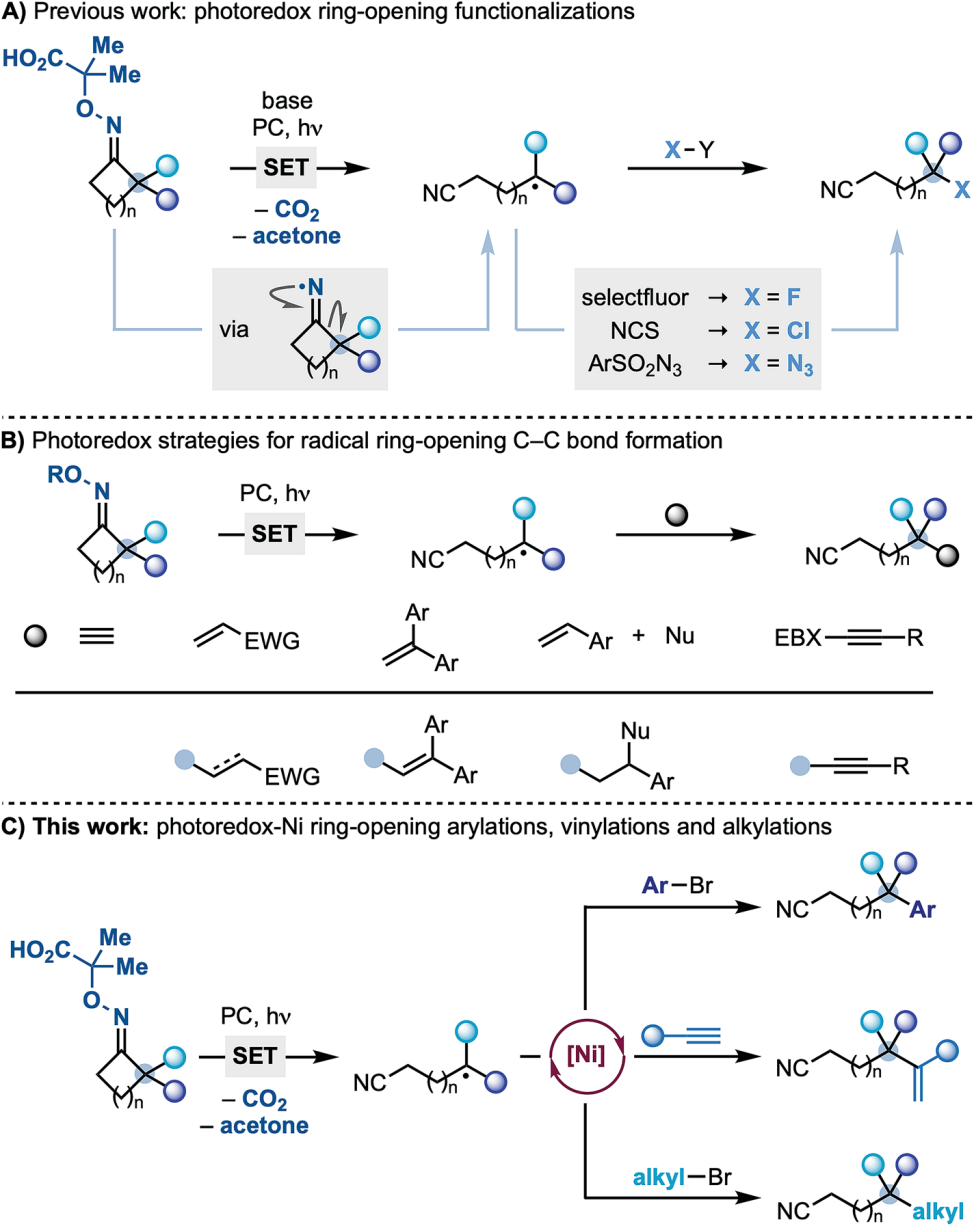
Strategies for the photo-induced ring-opening functionalizations of cyclic iminyl radicals. (A) Previous work: photoredox ring-opening functionalizations. (B) Photoredox strategies for radical ring-opening C–C bond formation. (C) This work: photoredox-Ni ring-opening arylations, vinylations and alkylations.

The Royal Society of Chemistry apologises for these errors and any consequent inconvenience to authors and readers.

